# PATZ1 Induces Apoptosis through PUMA in Glioblastoma

**DOI:** 10.1155/2022/4953107

**Published:** 2022-04-25

**Authors:** Xiang Tao, Ge Zhang, Junhui Liu, Baowei Ji, Haitao Xu, Zhibiao Chen

**Affiliations:** Department of Neurosurgery, Renmin Hospital of Wuhan University, Wuhan, 430060 Hubei Province, China

## Abstract

**Aim:**

This study was aimed at investigating the mechanism of PATZ1 inducing apoptosis through PUMA in glioblastoma. Overexpressed PATZ1 was transfected to explore its role in inducing apoptosis in glioblastoma cells.

**Methods:**

The expression of protein was detected by western blotting assay. qRT-PCR assay was used to detect the expression of RNA. Confocal microscopy was used to analyze the correlation between PATZ1 and PUMA. TUNEL assay was used to detect the cell apoptosis. The ability of cell proliferation was detected by MTT assay and EDU assay. The effects of PATZ1 on cell apoptosis and tumor proliferation were observed in vivo by tumor xenograft mouse model.

**Results:**

The results showed that low PATZ1 expression correlates with poor prognosis in glioblastoma patients. Overexpression of PATZ1 inhibits glioma cell proliferation and induces apoptosis by activating intrinsic apoptotic pathways. PATZ1 colocalizes intracellularly with PUMA inducing apoptosis through PUMA in glioblastoma.

**Conclusion:**

PATZ1 plays a biological regulatory role in inducing apoptosis in glioblastoma, and this regulatory effect is related to PUMA, and the specific mechanism remains to be further explored.

## 1. Introduction

Over 100 histological types exist in primary brain and central nervous system (CNS) tumors. CNS primary tumors account for only 2% of all primary cancers [1, 2]. Glioma is a broad term describing neuroepithelial tumors arising from glial or Sertoli cells of the CNS. Glial cells are the most abundant cell type in the central nervous system, and these glial cells surround and sequester neurons and provide nutrients and oxygen to neurons [3]. Epidemiology shows that malignant brain tumors occur frequently in men, and meningiomas and other nonmalignant tumors are more common in women [4–6].

The C2H2 zinc finger protein PATZ1 (POZ, AT-hook, and Zinc-finger 1), an important regulator of pluripotency in embryonic stem cells, is a member of the POK family and with SOX2 together to maintain self-renewal [7]. The POK family includes many transcription factors that play important roles in development, cell proliferation, senescence, and apoptosis [8]. The regulation of human tumors by PATZ1 depends on the cellular environment and sometimes acts as a tumor suppressor and sometimes as an oncogene [9]. PATZ1 has been shown to act as a prognostic factor in serous ovarian cancer, diffuse large B-cell lymphoma, and renal cell carcinoma [10]. In human brain tumor cells, PATZ1 has been found to be included in SOX2 interactome [11], and siRNA targeting of PATZ1 increased its sensitivity to apoptotic stimuli in human GBM cell lines resistant to conventional chemotherapy [12]. Previous studies have found that PATZ1 is a potential prognostic marker for adult GBM. High PATZ1 expression is associated with anterior neural subtypes and can predict better prognosis, while low expression is associated with interstitial subtype and predicts poor prognosis [13].

In the upregulation of apoptosis regulator (PUMA) by p53 which is a member of the Bcl-2 family present only in BH3 [14, 15], PUMA effectively induces mitochondrial permeability, cytochrome c release, and apoptosis by binding to other Bcl-2 family members, such as Bax, Bcl-2, and Bcl-xL [16, 17]. As a downstream target gene of p53, PUMA can induce apoptosis through a p53-dependent pathway, through which apoptosis caused by radiation, DNA-damaging drugs, hypoxia, and NO is achieved [18]. It has been shown that PUMA expression by p53-dependent pathway is the main mechanism for *γ*-ray-induced apoptosis of intestinal epithelial cells as well as hematopoietic stem cells [19]. p53 regulates PUMA expression at the transcriptional level when DNA damage occurs. However, PUMA can also induce apoptosis through a p53-independent pathway. Shaltouki et al. found that PUMA was able to directly mediate cytochrome c release and activate the aspartic enzyme cascade effect, allowing myoblasts to undergo apoptosis during skeletal muscle differentiation [20]. In addition, glucocorticoids induce lymphoid cells. PUMA is also involved in cellular processes [21, 22].

In this study, we found that downregulation of PATZ1 expression in glioma patients was associated with poor prognosis. PATZ1 overexpression was able to promote apoptosis of glioma cells. Therefore, we investigated whether PATZ1 could regulate PUMA. We further overexpressed PATZ1 and found that it could upregulate PUMA and promote its nuclear localization to induce apoptosis. In addition, we found that PATZ1 and PUMA interact and transcriptionally activate PUMA mRNA expression which leads to the activation of intrinsic apoptotic pathways. These results suggest that PATZ1 is a tumor suppressor and a potential target for therapeutic intervention in glioma.

## 2. Materials and Methods

### 2.1. Cell Culture and Transfection

Human cancer/noncancer cell lines U87-MG, U373, U343, U118-MG, and LN229 were purchased from Shanghai ATCC (Shanghai, China). U87-MG cells were cultured in MEM containing 10% fetal bovine serum (FBS) at 37°C in 5% CO_2_. U343, U373, U118-MG, and LN229 cells were all cultured in DMEM medium containing 10% FBS at 37°C in 5% CO_2_. PATZ1 overexpression plasmids were purchased from GenePharma (Shanghai, China). PATZ1 overexpression plasmids were transfected into U87-MG, U373, U343, U118-MG, and LN229 using Lipofectamine 2000 according to the instructions, and the expression of PATZ1 was determined by q-PCR assay.

### 2.2. Western Blot Assay

The cells were lysed in RIPA buffer, and total proteins were denatured and decomposed in 10-12% polyacrylamide gels and transferred to PVDF membranes for western blot analysis. PVDF membranes were blocked with 5% BSA in TBS and then incubated with primary antibodies overnight at 4°C. PVDF membranes were then incubated with horseradish peroxidase- (HRP-) conjugated secondary antibodies for 30 min at room temperature and then developed using enhanced chemiluminescence (ECL) (Millipore Company, USA).

### 2.3. RNA Extraction, cDNA Synthesis, and qRT-PCR

Total RNA was extracted from U343 and U87-MG cells using TRIzol reagent (Invitrogen, USA) according to the instructions, and a high-capacity RNA-to-cDNA kit (Takara, USA) was used for RNA reverse transcription. TaqMan Gene Expression Master Mix, Applied Biosystems 7900 Real-Time PCR Detection System (ABI 7900HT), was used to amplify each cDNA (2 *μ*L) in SYBR Green Real-Time PCR Master Mix (20 *μ*L) and finally quantify the expression of cDNA. The primers for this assay were PUMA: 3′-AGCAGCACTTAGTCGCC-5′/5′-CCTGGGTAAGGGGAGGTT-3′ and PATZ1: 5′-TACATCTGCCAGAGCTGTGG-3′/5′-TGCACCTGCTTGA TA TGTCC-3′; GAPDH primer sequence was 5′-GTCTCCTCTGACTTCAACAGCG-3′/5′-ACCACCCTG TTGCTGTAGCCAA-3′. Gene expression changes were measured using the 2(-Delta Delta C(T)) method [23].

### 2.4. MTT Assay

Cell viability was determined by MTT assay, and glioma cells in the logarithmic growth phase were seeded on 96-well plates at a density of 4 × 10^3^ cells/well. 20 *μ*L MTT solution (5 mg/mL, Sigma) was added to each well after 24 h and incubated for 4 h. Then 150 *μ*L dimethylsulfoxide (DMSO) (Sigma, USA) was added to each well. The cells were shaken at a low speed for 10 minutes to completely dissolve the crystals. The absorbance value was measured at OD 490 nm. The cells in the well which contained only the MTT and DMSO medium were used for the control group.

### 2.5. EDU Assay

U118-MG and U343 cells in the logarithmic growth phase were seeded on 96-well plates at a density of 4 × 10^3^ cells/well, and 100 *μL* BrdU (50 *μ*M) culture medium was added to each well after 24 h and incubated at room temperature for 2 h. Then, the cells were fixed in PBS solution containing 4% paraformaldehyde (PFA) for 30 min, 2 mg/mL glycine was added and incubated at room temperature for 5 min and then rinsed with PBS buffer and incubated with 1× Apollo staining reaction solution for 30 min in the dark, and then, the staining solution was discarded and washed with PBS containing 0.5% Triton X-100 for 10 min. PBS buffer was rinsed again and 1× Hoechst 33342 reaction solution was added for 30 min at room temperature in the dark. Finally, positive cells were observed using fluorescence microscopy (ThermoFisher Scientific, USA).

### 2.6. TUNEL Assay

Glioma cells in the logarithmic phase were seeded in 24-well plates with cell slides at a density of 10 [6] cells/well, and the cells were fixed with 4% PFA after 24 h and stained using a TUNEL apoptosis detection kit (Beyotime, Beijing, China) according to the instructions, mounted with DAPI staining solution and stained the nucleus, and finally observed under an inverted microscope (Olympus, Tokyo, Japan).

### 2.7. Immunofluorescence and Confocal Microscopy

For multilabel immunofluorescence, tissue immunosections were performed according to the procedure shown in the previous study [24]. After deparaffinization and hydration, first anti-PUMA (1 : 200 dilution) and anti-PATZ1 antibody (1 : 500 dilution) were incubated for 90 min at room temperature, and fluorescent secondary antibodies generated against different species (Alexa Fluor 594, with red signal, for rabbits, Alexa Fluor 488, with green signal, for mice) were used to localize antigen/primary antibody complexes. The dilution ratio of the second antibody was 1 : 100. Followed by DAPI covered slip and stained the nuclear. Finally, the observation of colocalization was performed and photographed under a confocal microscope (Olympus, Tokyo, Japan).

### 2.8. Tumor Xenograft Mouse Model

U118-MG cells at a density of 5 × 10^6^ cells transfected with PATZ1 or control vector were subcutaneously injected into each mouse. Tumor volumes were measured at 5-day intervals for 20 consecutive days. Experimental mice were euthanized and tumors were removed by dissection at the last day. Tumors were removed and fixed in 4% PFA. Tumor volume (m^2^) was calculated using the formula 1/2 × long diameter × short diameter. Tumor tissues were paraffin-embedded and sectioned after 72 hours of fixation. Tumor sections were observed for nuclear apoptosis by TUNEL apoptosis detection kit (Beyotime, Beijing, China). Finally, observation and analysis were performed under a fluorescence microscope (Olympus, Tokyo, Japan). The animal experiment was approved by the Ethics Committee of Renmin Hospital of Wuhan University.

### 2.9. Statistical Analysis

All data were expressed as mean ± SD. Statistical analysis was performed using SPSS 21.0 software. Statistical significance was calculated by Student's *t*-test or one-way analysis of variance. *P* < 0.05 was considered to be statistical significant. All experiments were performed at least three times.

## 3. Results

### 3.1. Low PATZ1 Expression Correlates with Poor Prognosis in Glioblastoma Patients

**Figure 1:**
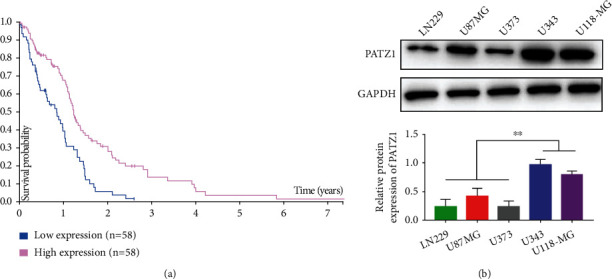
Low PATZ1 expression is associated with poor prognosis in GBM patients. (a) Kaplan-Meier survival curve of the PATZ1 expression in TCGA database. (b) Western blot analysis of PATZ1 expression in GBM cell lines. ^∗∗^*p* < 0.01.

In the TCGA database, glioblastoma patients were divided into two groups according to PATZ1 expression (according to median) (PATZ1 high expression group and PATZ1 low expression group), and the survival time of the two patients was analyzed. The Kaplan-Meier survival curve showed that the overall survival time of patients in PATZ1 low expression group was significantly shorter than that in PATZ1 high expression group (Figure 1(a)); western blot assay was used to investigate the PATZ1 expression of glioblastoma cell lines LN229, U87MG, U373, U343, and U118-MG; GAPDH was used as an internal reference. The results showed that in the above cell lines, the PATZ1 expression of U343 and U118-MG cell lines was significantly higher than that of LN229, U87MG, and U373 cell lines (Figure 1(b)), so U343 and U118-MG cell lines were used in this study.

### 3.2. Overexpression of PATZ1 Inhibits Glioma Cell Proliferation In Vitro

**Figure 2:**
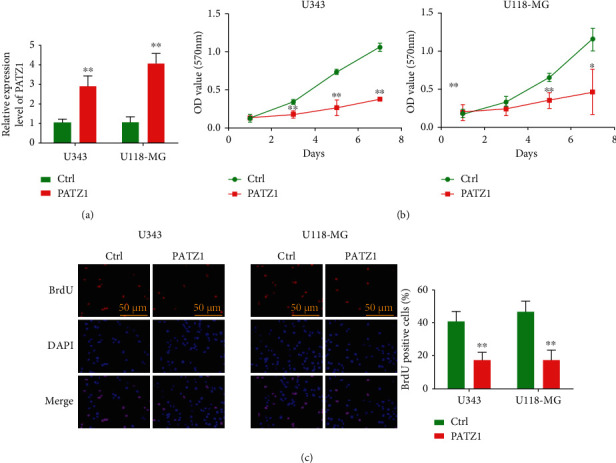
Overexpression of PATZ1 inhibits glioma cell proliferation in vitro. (a) qRT-PCR analysis of PATZ1 expression after PATZ1 overexpression in U343 and U118-MG cells. (b) Cell proliferation after overexpression of PATZ1 in U343 and U118-MG cells by MTT assay. (c) Cell proliferation after overexpression of PATZ1 in U343 and U118-MG cells by BrdU assay. ^∗^*p* < 0.05 and ^∗∗^*p* < 0.01.

qRT-PCR assay was performed after overexpression of PATZ1 in U343 and U118-MG cell lines, respectively; the results showed that in U343 cell line, the expression of PATZ1 in cells overexpressing PATZ1 group was significantly higher than that in the control group by plasmid transfection, and the same results could be observed in U118-MG cell line (Figure 2(a)); MTT was used to further detect the proliferation of the two cell lines after overexpression of PATZ1, and the results showed that overexpression of PATZ1 could significantly inhibit the proliferation of U343 cells and U118-MG cells (Figure 2(b)) in vitro; the results of BrdU assay also showed that after overexpression of PATZ1, the proliferation ability of U343 cells and U118-MG cells was significantly decreased; as shown in the figure, the number of positive cells in the overexpression group was significantly lower than that in the control group (Figure 2(c)).

### 3.3. Overexpression of PATZ1 Induces Apoptosis by Activating Intrinsic Apoptotic Pathways

**Figure 3:**
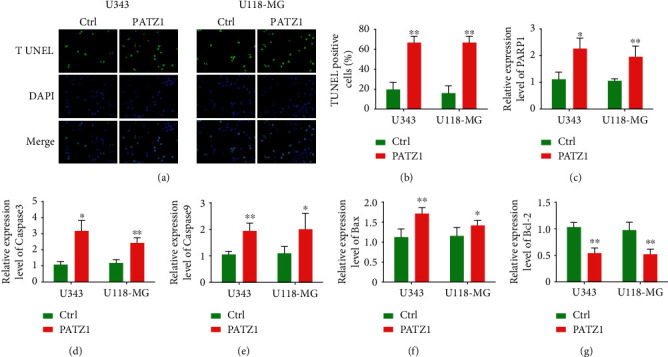
(a and b) TUNEL staining results after PATZ1 overexpression in U343 and U118-MG cells. (c–g) qRT-PCR results of PARP1, Caspase3, Caspase9, Bax, and Bcl-2 after overexpression of PATZ1 in U343 and U118-MG cells. ^∗^*p* < 0.05 and ^∗∗^*p* < 0.01.

TUNEL assay was used to detect apoptosis, and in U343 and U118-MG cell lines, apoptotic cells were significantly increased after overexpression of PATZ1 (Figure 3(a)); quantitative analysis revealed that the proportion of positive cells was significantly increased in the overexpression group, respectively (Figure 3(b)), and the expression of apoptosis-related genes was further compared; qRT-PCR results showed that after the overexpression of PATZ1 in U343 and U118-MG cell lines, the expression of PARP1, Caspase3, Caspase9, and Bax was significantly increased (Figures 3(c)–3(f)), while the expression of Bcl-2 was significantly decreased with the overexpression of PATZ1 Figure 3(g)), indicating that after the overexpression of PATZ1, the intrinsic apoptosis-related pathway was activated, thereby inducing apoptosis.

### 3.4. Apoptosis Induced by PATZ1 Requires PUMA Involvement

**Figure 4:**
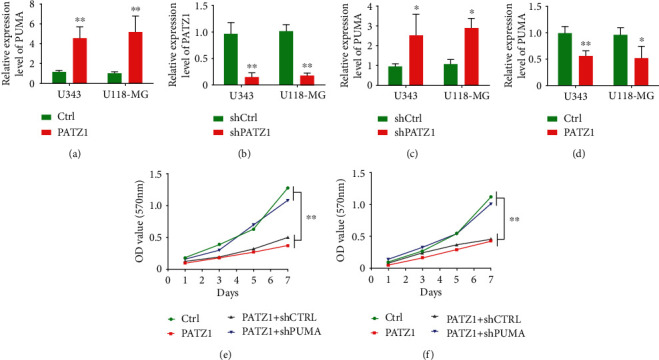
PATZ1-induced apoptosis requires PUMA. (a) RT-PCR results of mRNA expression of PUMA after overexpression of PATZ1 in U343 and U118-MG cells. (b) RT-PCR results of mRNA expression of PATZ1 after knockdown of PATZ1 in U343 and U118-MG cells. (c) RT-PCR results of mRNA expression of PUMA after knockdown of PATZ1 in U343 and U118-MG cells. (d) RT-PCR results of mRNA expression of PUMA after knockdown of PUMA in U343 and U118-MG cells. (e) MTT results of different treatment groups in U343 cells. (f) MTT results of different treatment groups in U118-MG cells. ^∗^*p* < 0.05 and ^∗∗^*p* < 0.01.

To further explore the mechanism of PATZ1-induced apoptosis, we overexpressed PATZ1 and extracted total RNA in U343 and U118-MG cells. RT-PCR results showed that the mRNA expression of PUMA increased with PATZ1 expression increasing (Figure 4(a)); total RNA was extracted after further knockdown of PATZ1, and RT-PCR analysis showed that PATZ1 expression was significantly decreased in both cell lines (Figure 4(b)), and the mRNA level of PUMA was significantly decreased (Figure 4(c)), indicating that the level of PUMA changed with the change of PATZ1 expression. Further, PUMA was knocked down in the two cell lines, and RT-PCR results showed that PUMA expression was significantly decreased (Figure 4(d)); in order to further verify the regulatory relationship of PATZ1 on PUMA, we divided the cells into four groups, which were Ctrl, PATZ1, PATZ1+shCtrl, and PATZ1+shPUMA, and detected the cell proliferation of different transfection treatment groups by MTT assay. The results showed that in the U343 cell line, the cell proliferation rate of the group overexpressing PATZ1 was significantly decreased compared with the Ctrl group, and there was no significant difference between the PATZ1+shCtrl group and the PATZ1 group, but the cell proliferation rate of the PATZ1+shPUMA group was significantly increased compared with the PATZ1+shCtrl group (Figure 4(e)); the same results were observed in the U118-MG cell line (Figure 4(f)).

### 3.5. PUMA Colocalizes Intracellularly with PATZ1

**Figure 5:**
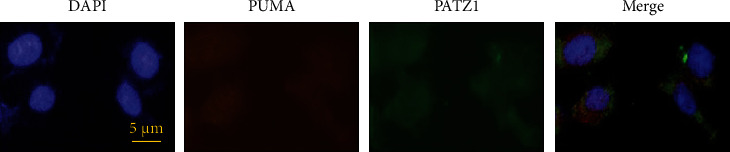
PUMA colocalizes with PATZ1 in U118-MG cells.

Immunofluorescence assays were used to further verify the relationship between the two proteins, which we performed in U118-MG cells, and the results showed that the two proteins colocalized well in the cells (Figure 5), thus further verifying the biological relationship between the two proteins.

### 3.6. PATZ1 Inhibits Tumor Growth and Induces Apoptosis In Vivo

**Figure 6:**
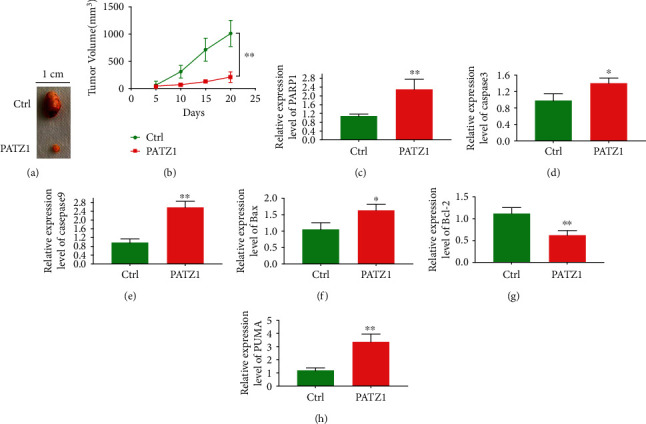
PATZ1 inhibits tumor growth and induces apoptosis in vivo. (a) Tumors in mice overexpressing PATZ1. (b) Tumor growth curve in mice overexpressing PATZ1. (c–h) Expression of PARP1, Caspase3, Caspase9, Bax, Bcl-2, and PUMA in tumor tissues of mice overexpressing PATZ1. ^∗^*p* < 0.05 and ^∗∗^*p* < 0.01.

The xenograft model further verified the effects of PATZ1 on tumor growth and tumor cell proliferation in vitro. U118-MG cells overexpressing PATZ1 were subcutaneously injected into nude mice and dissected on the 20th day after tumor bearing. The tumor growth curve showed that the tumor volume in the overexpression group was significantly smaller than that in the Ctrl group (Figures 6(a) and 6(b)); the apoptosis-related genes in the tissues were further detected. The qRT-PCR results showed that the expressions of PARP1, Caspase3, Caspase9, and Bax in the tumor tissues of mice overexpressing PATZ1 were significantly increased (Figures 6(c)–6(f)), but the levels of Bcl-2 were decreased with the overexpression of PATZ1 (Figure 6(g)). At the same time, in mice overexpressing PATZ1, the expression of PUMA was also increased (Figure 6(h)), indicating that PATZ1 can also influence the expression of PUMA in vivo, induce tumor cell apoptosis, and thus inhibit tumor growth.

## 4. Discussion

GBM is the most common central nervous system malignancies, accounting for 12%–15% of all intracranial tumors and approximately 45.6% of primary malignant brain tumors [25, 26]. GBM has a poor prognosis with a 5-year survival rate of less than 10% [27], and one of the important reasons for this is that highly aggressive brain tumors are resistant to radiotherapy and chemotherapy. When the pathway controlling apoptosis is changed in glioblastoma cells, it can lead to cell resistance to apoptosis. In the current study, alterations in the p53 pathway, BCL-2 protein family, inhibitor of apoptosis protein, and several growth factor pathways involved in the regulation of programmed cell apoptosis have been identified, further providing possible targets for new therapies based on these apoptotic pathways in GBM. The process of apoptosis includes intracellular hypoxia, DNA damage, cell cycle defects, or loss of cell survival factors, which significantly correlate with the activated of caspase-3, Bcl-2, and Bcl-2 associated antiapoptotic protein Bax. Therefore, the search for key factors regulating autophagy and apoptosis may be a critical step in the treatment of glioblastoma.

It has been reported that *β*1 4-galactosyltransferase I (B4GalT1) expression is significantly increased in GBM and plays a significant role in inflammatory activation and regulation of apoptosis [28], and other studies have reported that SB365 can inhibit the growth of GBM cells and induce morphological characteristics of cell tendency to apoptosis, such as nuclear condensation and fragmentation, enhanced expression of caspase-3, and significantly delayed cell migration and decreased HIF-1*α* expression and VEGF secretion [29]. In this study, we found that PATZ1 can regulate the apoptotic process of GBM and affect the malignant development of tumor cells in vivo and in vitro, and past reports have also confirmed the high expression status of PATZ1 in GBM, especially for proneural GBM subtypes, showing significant enrichment [13], and recent studies have pointed out that PATZ1 is a multifunctional regulator of embryonic stem cells and is directly involved in the regulation of key genes, such as Pou5f1 and Nanog, to cooperate with sox2 to maintain cell self-renewal [7]; some studies have also proposed a role for PATZ1 in neural stem cells, and knockdown of PATZ1 can bring about proliferation defects [30]; more importantly, PATZ1 is a member of the POK family, a unique group of transcription factors, which play a key role in cell development and cancer development by participating in various cellular processes, including cell proliferation, senescence, and apoptosis [8, 12, 31–33], which is basically consistent with our findings. In this study, PATZ1 can be involved in regulating the expression of apoptosis-related factors, such as promoting the expression of apoptosis-related classical genes PARP1, caspase 3, caspase 9, and Bax and inhibiting the expression of Bcl-2, directly or indirectly inducing apoptosis in GBM tumor cells.

PUMA is a proapoptotic Bcl2 family protein, which can be transferred from cytosol to mitochondria to induce apoptosis. It has been pointed out that PUMA can activate Bax/Bak to exert its role in regulating apoptosis under conditions such as oxidative stress, DNA damage, and hypoxia [34, 35]. It can directly activate the proapoptotic proteins Bax and Bak via the BH3 domain, thereby inducing mitochondrial dysfunction. Alternatively, it can regulate apoptosis by indirectly releasing Bax and Bak through interaction with antiapoptotic Bcl-2 family proteins such as Bcl-xL and Bcl-2 [36, 37]. This study found that the overexpression of PATZ1 can promote apoptosis. PUMA can regulate mitochondrial membrane permeability, proapoptotic molecule release, and Caspase cascade effect. Therefore, we investigated whether PATZ1 could regulate PUMA. In this study, we found that PATZ1 could achieve good colocalization with PUMA and preliminarily verified the synergistic effect of PATZ1 and PUMA in the knockdown PUMA group. After knockdown of PUMA in the presence of overexpression, the apoptosis-inducing effect of PATZ1 was reduced. Similarly, in vitro studies, the overexpression status of PATZ1 also affected PUMA expression in tumor tissues, indicating that PUMA plays a direct or indirect synergistic or regulatory relationship in the apoptotic regulation process of PATZ1, but the specific regulatory or synergistic mechanism has not been thoroughly elucidated and needs further study.

## 5. Conclusions

In GBM, PATZ1 plays a biological regulatory role in inducing apoptosis, and this regulatory effect is related to PUMA, and the specific mechanism remains to be further explored.

## Data Availability

The data used to support the findings of this study are available from the corresponding author upon request.
